# Exploring Factors Influencing Driving Simulator Performance in Patients With Acquired Brain Injury Using Hierarchical Clustering Analysis of Principal Components

**DOI:** 10.7759/cureus.82557

**Published:** 2025-04-19

**Authors:** Shuto Takehara, Tasuku Sotokawa, Yuta Tauchi, Toshiaki Sato, Rie Sakamoto, Yoshihiro Kanata, Kazuhisa Domen

**Affiliations:** 1 Department of Rehabilitation Medicine, Hyogo Medical University Sasayama Medical Center, Hyogo, JPN; 2 Graduate School of Health Sciences, Yamagata Prefectural University of Health Sciences, Yamagata, JPN; 3 Department of Occupational Therapy, Faculty of Health Science, Yamagata Prefectural University of Health Sciences, Yamagata, JPN; 4 Department of Rehabilitationl Medicine, Hyogo Medical University Sasayama Medical Center, Hyogo, JPN; 5 Department of General Medicine and Community Health, Hyogo Medical University Sasayama Medical Center, Hyogo, JPN; 6 Department of Rehabilitation Medicine, Hyogo Medical University School of Medicine, Hyogo, JPN

**Keywords:** acquired brain injury, driving rehabilitation, driving simulator, motor function, multiple factor analysis, neuropsychological test, principal components clustering, stroke, training effects, traumatic brain injury

## Abstract

Background

Driving simulator training is widely recognized as an effective tool for driving rehabilitation. However, the key factors influencing simulator performance and the extent of training-related improvements remain insufficiently explored. This study aimed to identify the demographic, motor, and cognitive factors associated with driving simulator performance and post-training improvements in patients with acquired brain injury (ABI) using clustering analysis.

Methods

A total of 64 patients with ABI (59% cerebral hemorrhage, 34% cerebral infarction, 7% traumatic brain injury; mean age 64±13 years; 81% male) underwent comprehensive neuropsychological assessments and driving simulator evaluations before and after training. Multiple factor analysis was applied to integrate pre- and post-training variables and reduce dimensionality. Hierarchical Clustering on Principal Components was then performed to classify patients based on training effect patterns. The Kruskal-Wallis test and post hoc multiple comparisons were used to assess differences in background factors among the clusters.

Results

Three distinct clusters were identified: Cluster 1 (n=32) exhibited consistently high performance in reaction and city-driving tasks, Cluster 2 (n=19) demonstrated prolonged reaction times but showed significant improvements in city-driving tasks after training, and Cluster 3 (n=13) demonstrated severe city-driving errors and limited post-training improvement. Neuropsychological assessments revealed significant differences among the clusters (p < 0.05), with Cluster 1 consistently outperforming Clusters 2 and 3 across multiple cognitive domains, including attention, cognitive flexibility, visuospatial abilities, memory, and executive function.

Conclusion

Neuropsychological assessments may serve as predictors of both baseline driving performance and post-training improvements. Tailoring interventions to individual cognitive profiles, particularly focusing on attention, visuospatial abilities, and executive function, may enhance the efficacy of simulator-based rehabilitation and support the safe resumption of driving. Future longitudinal studies should examine how targeted cognitive training might improve driving performance in patients with different cognitive profiles.

## Introduction

Driving allows individuals to travel beyond walking distance and accomplish daily goals such as commuting, shopping, and attending medical appointments [1]. For many individuals, particularly those with acquired brain injury (ABI), driving is essential not only for maintaining daily life and fulfilling occupational needs but also for personal identity and autonomy [2-3]. The loss of driving ability following ABI can have significant negative impacts, including increased rates of depression, reduced confidence and independence, and diminished social participation [4-6]. Therefore, supporting individuals with ABI in resuming driving safely is an important rehabilitation goal that requires effective assessment and training approaches.

In clinical practice, driving simulators offer a dual functionality that makes them particularly valuable in rehabilitation of patients with ABI. As an assessment tool, simulators enable clinicians to objectively measure reaction time, hazard perception, and decision-making across various traffic scenarios in a safe and controlled environment [7-8]. As a training device, simulators have demonstrated remarkable efficacy, leading to better-maintained improvements in real-car assessments after six months for patients with stroke [9], reductions in dangerous driving behaviors in patients with traumatic brain injury [10], and increased driving confidence in mixed populations of patients with stroke and traumatic brain injury [11]. Despite these advantages, studies have reported considerable variations in training protocols, from brief sessions over a few weeks to extensive programs spanning several months [10-15], contributing to heterogeneity in findings.

Research has explored various aspects of driving simulator performance, including the impact of stroke lesion location [16], differences between left and right hemisphere injuries [17], and the relationship between executive dysfunction and driving performance [18]. However, the factors influencing simulator performance, particularly their associations with cognitive and functional assessments, remain insufficiently explored. Specifically, the relationship between demographic factors, such as age [19], sex [20], time since injury onset [21], and the effects of stroke versus TBI [22], and driving simulator performance has not been fully investigated, even though these factors are known to influence general cognitive function assessments. Additionally, it remains unclear which cognitive domains most strongly predict both baseline driving ability and the capacity to improve with simulator training.

This study aimed to identify the demographic, motor, and cognitive factors that influence driving simulator performance and the extent of improvements following training. We hypothesized that patients with stronger cognitive profiles, particularly in domains of attention, executive function, and visuospatial abilities, would demonstrate both better baseline driving performance and greater post-training improvements. To systematically identify patterns among these factors, we employed clustering analysis, which allows for the classification of patients based on similar performance characteristics and response to training [23]. Understanding these associations may help clinicians tailor and optimize intervention strategies.

## Materials and methods

Study design and settings

This case-control study utilized data from patients assessed for resumption of driving at the University of Hyogo Sasayama Medical Center between October 1, 2017, and August 31, 2024. This extended time frame was selected to ensure a sufficient sample size for the clustering analysis. The study adhered to the Strengthening of the Reporting of Observational Studies in Epidemiology (STROBE) guidelines.

Participants

The study population included inpatients and outpatients diagnosed with either stroke or traumatic brain injury who underwent driving assessments at the hospital during the study period. To be eligible for inclusion, patients were required to demonstrate at least moderate independence in their daily activities, defined as a score of ≥6 on the motor items of the Functional Independence Measure (FIM) [24], excluding bathing. In addition, participants needed to have sufficient comprehension and communication skills, with a score of ≥6 on the comprehension and expression items of the FIM. Only individuals aged ≥18 years were included. The exclusion criteria were as follows: a history of other neurological disorders, a clinical diagnosis of epilepsy rendering them ineligible for a driving license under the Japanese Road Traffic Law, or visual field disorders such as homonymous hemianopsia or quadrantanopia. Further exclusion criteria included a history of multiple strokes or traumatic brain injuries and multiple lesion sites. These criteria were established to examine the effects of a single brain injury without confounding factors from other conditions. Additional exclusion criteria included withdrawal from the evaluation process due to simulator sickness (as simulator sickness prevents adequate performance assessment) and missing data (to avoid potential bias in the analysis).

This study was approved by the Ethical Review Committee of Hyogo Medical University (No. 4464) and was conducted in accordance with the Declaration of Helsinki. A dedicated website was created to provide additional study details, including an opt-out option for participants.

Study procedure

All participants underwent one motor function evaluation and eight cognitive function evaluations. These assessments were followed by driving simulator-based evaluations conducted in two phases: pre-training and post-training. In both phases, participants completed two reaction tasks and a city-driving course using a driving simulator. To ensure a clear understanding of the tasks, the operation of the simulator and the assessment procedures were thoroughly explained before testing. Following the pre-training assessments, participants engaged in a structured training program designed to improve their driving performance. These sessions were conducted across three levels of difficulty (beginner, intermediate, and advanced) and aimed at progressively improving driving skills through simulated city-driving courses.

Training session

The training program comprised nine sessions, divided into three levels: beginner, intermediate, and advanced. Each level consisted of three sessions using the driving simulator, where participants practiced navigating increasingly complex driving environments. Every session was supervised by an occupational therapist certified as a Driving and Community Mobility Practitioner by the Japan Association of Occupational Therapists. The therapist provided real-time guidance during the sessions and delivered detailed post-session feedback. Feedback sessions were conducted after each training session and lasted approximately 5-10 minutes. Using the simulator's replay function and performance data, they highlighted areas for improvement, emphasizing reaction times, adherence to traffic rules, and overall driving techniques. Before commencing the training, the operation of the simulator and the specific task requirements were carefully explained to all participants. This preparatory step was essential for maintaining consistency in assessments and maximizing the effectiveness of the training.

Measurements

Patient data, including demographic information, motor function, cognitive function, and driving simulator performance, were collected from the Hyogo Medical University Sasayama Medical Center.

Motor function

Motor function in patients with stroke was assessed using the Brunnstrom Recovery Stage (BRS), which evaluates motor recovery across three categories: upper extremity, hand/finger, and lower extremity. The BRS classifies motor recovery into six stages, with higher stages indicating better motor function and improved recovery [25].

Cognitive function

Cognitive function was assessed using eight neuropsychological tests, all administered in a paper-and-pencil format: the Mini-Mental State Examination Japanese Version (MMSE-J) [26], Trail Making Test Japanese Version (TMT-J), including Part A and Part B [27], Behavioural Inattention Test Conventional Subtests (BITC) [28], Kohs Block-Design Test (KBDT) [29], Rey-Osterrieth Complex Figure Test (ROCFT) [30], Rivermead Behavioral Memory Test (RBMT) [31], Behavioral Assessment of the Dysexecutive Syndrome (BADS) [32], and Stroke Drivers' Screening Assessment Japanese Version (J-SDSA) [33].

Apparatus and driving simulator task

**Figure 1:**
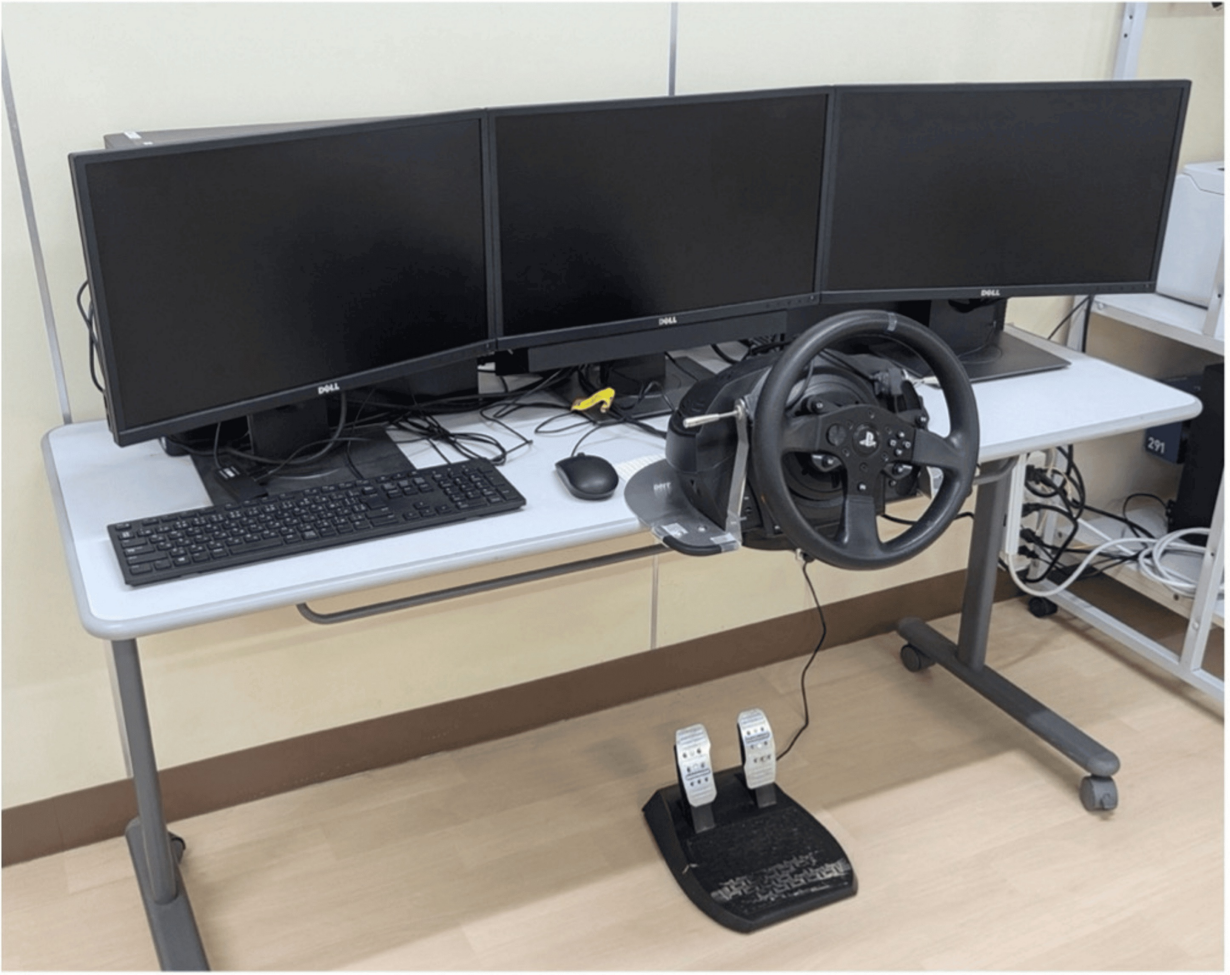
Overview of Honda’s Safety Navi® The driving simulator used in this study.

The driving simulator task consisted of a simple reaction task, a selective reaction task, and a city-driving course displayed on three screens (Figure 1). The patient sat approximately 75 cm from the monitor. The steering wheel was fixed to the table in front of the patient, and a pedal unit consisting of a brake and an accelerator was placed at the patient’s feet. The left and right displays were arranged in a trapezoidal shape, with a viewing angle of 65 ± 5°. The speedometer, accelerator pedal scale, and brake pedal scale were projected onto the lower part of the central display.

In both the simple and selective reaction tasks, patients were required to perform maneuvers as quickly as possible. During these tasks, participants were instructed to press the accelerator pedal fully (100%) to the floor while maintaining a simulated driving speed of 40 km/h. If the accelerator pedal was depressed by <90%, the monitor remained unilluminated. In the simple reaction task, the driver released the accelerator pedal when the green lamp of the vehicle was illuminated (Appendix A). In the selective reaction task, the driver was required to respond to the red (Appendix B), yellow (Appendix C), and blue lamps (Appendix D) of the vehicle in front. When the red lamp was illuminated, the driver had to immediately press the brake pedal after releasing the accelerator pedal. When the yellow lamp appeared, the driver needed to release the accelerator pedal. When the blue lamp was illuminated, the driver was instructed to maintain pressure on the accelerator pedal.

The variables that were assessed in the simple and selective reaction tasks included mean reaction time, standard deviation (SD) of reaction time, and number of false reactions in the simple reaction task. In the selective reaction task, the variables included mean red lamp reaction time, SD of red lamp reaction time, mean yellow lamp reaction time, SD of yellow lamp reaction time, and number of false reactions.

The city-driving course required participants to recognize hazardous situations in mixed traffic, predict potential hazards, and exhibit appropriate driving behaviors to avoid accidents in a simulated city center (Appendix E). The course consisted of three variations (courses 1, 2, and 3), with course 1 used in this study. Performance in the city-driving course was assessed using 20 criteria: sudden acceleration, ignoring a stop sign, sudden braking, successful parking, failure to use turn signals, incorrect use of turn signals, inadequate rear observation, failure to observe the road ahead, disregarding traffic signals and road signs, improper halt, improper lane positioning, unsafe following and lateral distances, improper lane positioning during merging and cornering, speeding percentage on the course, average excess speed, average turning speed at intersections, route deviation, missed instructional signs, near collision within 1 m, and accidents. Detailed definitions and measurement criteria for these indicators are provided in Table 1. The simulated road design followed a left-hand traffic system in accordance with Japanese Road Traffic Law.

**Table 1:** Definitions and measurement criteria for city-driving course performance indicators

Performance indicator	Definition and measurement criteria
Sudden acceleration	Immediately after the start of the journey and approximately 10 m before the stop line, if there is a change in the accelerator (degree of depression) of >50% within 0.1 s (indicating excessive accelerator depression).
Ignoring a stop sign	The vehicle did not come to a complete stop (speed remained > 0.1 km/h) approximately 10 m before the stop line and upon reaching the stop line.
Sudden braking	A rapid increase in brake application (pedal depression) of >50% within 0.1 s, occurring between approximately 10 m before the stop line and the point at which the vehicle reaches the stop line.
Successful parking	The vehicle reached a complete stop (speed was < 0.1 km/h) within the designated parking space boundaries.
Failure to use turn signals	Neither the left nor right signals were activated when required.
Incorrect use of turn signals	The wrong turn signal was activated at a location where signaling was necessary.
Inadequate rear observation	The distance between the designated vehicle and the rear of the vehicle (rear bumper) was less than the required minimum (approximately 1.0 m).
Failure to observe the road ahead	The distance between the designated vehicle or object and the front bumper of the vehicle was less than the required minimum (approximately 1.0 m).
Disregarding traffic signals and road signs	The number of instances where one or more of the following conditions occurred in the city-driving task: (ⅰ) the vehicle proceeded through a red traffic light, (ii) the speed did not decrease to <0.1 km/h (stop) within approximately 10 m before reaching the stop line, (ⅲ) the vehicle took the wrong course contradicting an information sign.
Improper halt	The driver crossed the intersection without stopping (at a speed of <1.0 km/h) when the traffic light in the direction of travel was red or at a location where a stop was required.
Improper lane positioning	Part of the vehicle extended beyond the center lane within the designated limits.
Unsafe following and lateral distances	The distance between the vehicle and the vehicle ahead, or the lateral distance when passing a stationary vehicle, was less than the required minimum (approximately 1.0 m).
Improper lane positioning during merging and cornering	The vehicle crossed the centerline within the designated limits while merging onto the main road or navigating a curve.
Speeding percentage on the course	The proportion of the total driving distance during which the vehicle exceeded the posted speed limit.
Average excess speed	The average amount by which the vehicle exceeded the speed limit in areas where speeding occurred.
Average turning speed at intersections	The average speed while turning right and left at intersections.
Route deviation	The number of times in which the vehicle strayed from the correct course.
Missed instructional signs	A course error occurred at an instructional sign.
Near collision within 1 m	The distance between the designated vehicle and another vehicle was less than the required minimum (approximately 1.0 m).
Accidents	The number of collisions that occurred between the start and end of the city-driving course.

Statistical analysis

Patient characteristics were summarized using medians and interquartile ranges for continuous variables, whereas categorical variables were presented as frequencies and percentages. Participants with any missing values in key measurements were excluded from the analysis to ensure complete datasets for the clustering approach. This study aimed to assess the effects of driving simulator training by analyzing changes in variables between pre- and post-training assessments. To achieve this, a two-step approach was employed within a common-dimensional framework. First, a multiple factor analysis (MFA) was applied to integrate the pre- and post-training data, reducing the dimensionality of multiple simulator-based variables. This method enabled the consolidation of correlated groups of variables, such as those related to reaction time tasks and city-driving performance, into unified dimensions. Subsequently, hierarchical clustering on principal components (HCPC) was performed to identify distinct patterns of training effects, referred to as clusters [34].

MFA was chosen over standard principal component analysis because it allows for the integration of pre- and post-training variables while maintaining their relational structure. The MFA approach was applied to the driving simulator data from pre- and post-training assessments, which inherently standardizes variables within each assessment group (pre- and post-training), accounting for different measurement scales across various driving simulator metrics such as reaction times, error counts, and speed measurements. In this study, variables were categorized into blocks based on the pre- and post-training phases. This approach provided a more comprehensive understanding of patterns across multiple groups, minimized information loss, and facilitated the accurate identification of diverse training effect patterns. A total of 112 variables were collected from the driving simulator data, spanning both pre- and post-training assessments. These variables were classified into eight groups based on outputs from the city-driving course: simple reaction task, selective reaction task, start/stop behavior, signaling, safety checks, positioning, speed, and overall driving performance. For the clustering analysis, performance scoring for each variable followed the standardized measurement criteria detailed in Table 1, with each indicator being measured according to its specific operational definition (e.g., reaction times in seconds, error counts as frequencies, speeding as percentages). Each group was treated as a separate block within the MFA framework.

In the first step, MFA was applied to extract the most informative dimensions from the dataset. The selection of dimensions was based on eigenvalues > 1, the identification of an elbow point in the scree plot (indicating a sharp change in the explained variance), and the interpretability of the MFA results [35]. In the second step, HCPC was used to perform hierarchical clustering on the retained principal components. The number of clusters was determined using the automatic clustering approach (nb.clust=-1) in the HCPC algorithm, which identifies the optimal number of clusters by analyzing the inertia gain. This method examines the dendrogram structure and identifies the point where further subdivision would result in minimal information gain. HCPC was initially performed using hierarchical clustering, with cluster assignments subsequently refined using the k-means algorithm with a maximum of 20 iterations (iter.max=20).

To interpret the cluster characteristics, v-tests were conducted to determine the relevance of differences between cluster-specific variable values and the overall mean. Higher v-test values indicated greater importance of a variable in defining a specific cluster [36]. Positive v-test values signified that the mean within the cluster was higher than the overall mean, whereas negative values indicate a lower mean. A v-test value > |1.96| corresponded to a p-value < 0.05, signifying statistical significance.

After clustering, background factors among the clusters were compared using the Kruskal-Wallis test, followed by post hoc multiple comparison tests. Multiple comparisons were adjusted using the Bonferroni method to control for type I errors. Fisher's exact test was employed for the analysis of categorical variables. A significance level of 5% was set for all statistical tests. Data analyses were performed using R software (version 4.2.2, R Foundation for Statistical Computing, Vienna, Austria) and the FactoMineR package [34].

## Results

**Figure 2:**
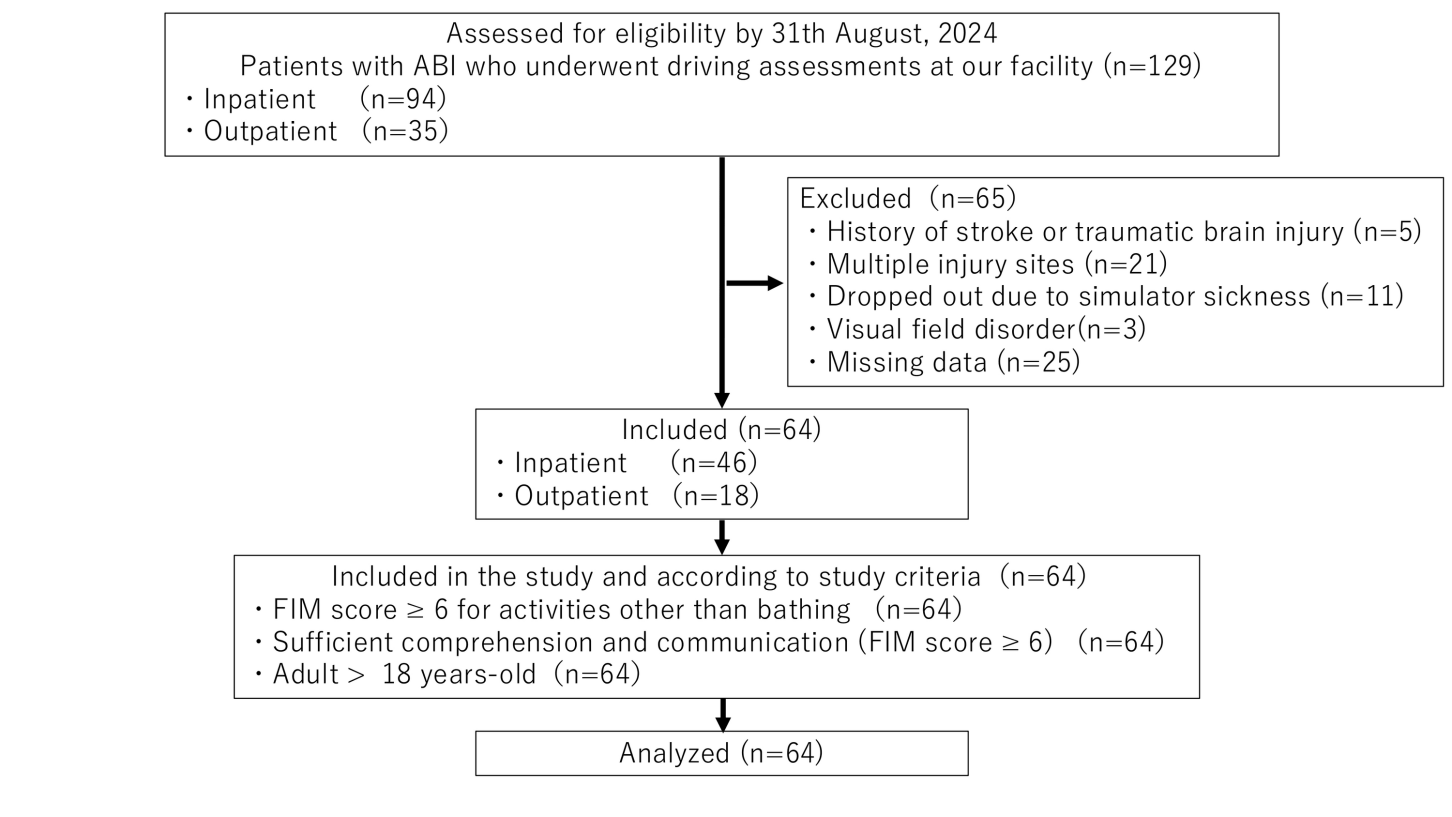
Patient flowchart. ABI, acquired brain injury; FIM, Functional Independence Measure

Between October 1, 2017, and August 31, 2024, a total of 129 patients with ABI underwent driving assessments at our institution. After applying the exclusion criteria, 65 patients were excluded, leaving 64 patients eligible for cluster analysis (Figure 2).

Demographic characteristics of study participants

The mean age of the patients was 64 years, with 81% being male. The most common diagnosis was cerebral hemorrhage, as observed in 38 patients. The lesions were almost equally distributed between the right and left hemispheres. The average time from onset to the pre-assessment was 133 days, with a nine-day interval between pre- and post-assessments. Regarding functional and cognitive assessments, the mean FIM score was 122, and the median BRS score was 6. The average score on the MMSE-J was 28. The mean times recorded for the TMT-J Part A and Part B were 77 s and 129 s, respectively. The mean total score for the BITC was 142, whereas the mean KBDT IQ score was 89. In addition, the mean score for the ROCFT was 33.55, the mean total profile score for the RBMT was 19, and the mean total profile score for the BADS was 16. Most patients successfully passed the J-SDSA (Table 2).

**Table 2:** Demographic characteristics of the study participants. Date are presented as mean ± SD, n (%), or median (IQR) FIM, Functional Independence Measure; BRS, Brunnstrom Recovery Stage; MMSE-J, Mini-Mental State Examination Japanese Version; TMT-J Part A and Part B, Trail Making Test Part A and Part B Japanese Version; BITC, Behavioral Inattention Test Conventional Subtests; KBDT, Kohs Block Design Test; ROCFT, Rey–Osterrieth Complex Figure Test; RBMT, Rivermead Behavioral Memory Test; BADS, Behavioral Assessment of the Dysexecutive Syndrome; J-SDSA, Stroke Drivers’ Screening Assessment Japanese Version; SD, standard deviation; IQR, interquartile range

Variable	n = 64
Age (years)	64 ± 13
Sex	
Female	12 (19%)
Male	52 (81%)
Diagnosis	
Cerebral infarction	22 (34%)
Cerebral hemorrhage	38 (59%)
Traumatic brain injury	4 (7%)
Lesion side	
Right	30 (47%)
Left	34 (53%)
Onset-to-assessment time (days)	133 (32–267)
Interval between pre- and post-assessments (days)	9 (5–16)
FIM	
Motor function score	88 ± 3
Cognitive function score	34 ± 2
Total score	122 ± 4
BRS	
Upper extremity	6 (5–6)
Hand/fingers	6 (6–6)
Lower extremity	6 (6–6)
MMSE-J total score	28 ± 2
TMT-J Part A (s)	77 ± 36
TMT-J Part B (s)	129 ± 65
BITC total score	142 ± 4
KBDT IQ score	89 ± 19
ROCFT copy score	33.55 ± 3.42
RBMT total profile score	19 ± 4
BADS total score	16 ± 4
J-SDSA prediction of fitness to drive	
Fail	19 (30%)
Pass	45 (70%)

Pre- and post-performance on the driving simulator

Table 3 presents a comparison of the pre- and post-training performance on the driving simulator for the 64 study participants. In the simple reaction task, both the reaction time (p < 0.001) and its SD (p < 0.001) showed significant reductions after training. In the selective reaction task, post-training results demonstrated significant reductions in reaction times for both the red (p < 0.001) and yellow lamps (p < 0.001) compared to the pre-training results. In addition, the SDs of the reaction times for the red (p = 0.005) and yellow lamps (p = 0.003), as well as the number of false reactions (p < 0.001), were significantly reduced after the training.

**Table 3:** Results of pre- and post-performances on the driving simulator (n = 64). ^a^Mean ± SD; ^b^Paired t-test CI, confidence interval; SD, standard deviation

Variable	Pre-performance, n = 64^a^	Post-performance, n = 64^a^	Difference^b^	95% CI^b^	t-Value^b^	p-Value^b^
Simple reaction task						
Reaction time (s)	0.46 ± 0.10	0.42 ± 0.07	0.03	0.02–0.05	4.06	<0.001
Reaction time SD (s)	0.12 ± 0.08	0.08 ± 0.05	0.03	0.01–0.05	3.48	<0.001
False reaction (times)	0.81 ± 1.88	0.63 ± 2.10	0.19	-0.53 to 0.90	0.52	0.6
Selective reaction task						
Red lamp reaction time (s)	1.09 ± 0.24	0.98 ± 0.23	0.11	0.05–0.17	3.88	<0.001
Red lamp reaction time SD (s)	0.14 ± 0.06	0.11 ± 0.05	0.03	0.01–0.05	2.92	0.005
Yellow lamp reaction time (s)	0.80 ± 0.18	0.72 ± 0.13	0.08	0.05–0.10	5.11	<0.001
Yellow lamp reaction time SD (s)	0.18 ± 0.08	0.15 ± 0.06	0.03	0.01–0.05	3.11	0.003
False reaction (times)	7 ± 9	3 ± 3	3.9	1.7–6.0	3.61	<0.001
City-driving course						
Sudden acceleration (times)	0.22 ± 0.42	0.30 ± 0.55	-0.08	-0.23 to 0.07	-1.04	0.3
Ignoring a stop sign (times)	1.66 ± 1.39	1.02 ± 1.19	0.64	0.27–1.0	3.45	<0.001
Sudden braking (times)	0.75 ± 1.04	0.38 ± 0.65	0.38	0.11–0.64	2.87	0.006
Successful parking (times)	0.38 ± 0.49	0.58 ± 0.50	-0.20	-0.36 to -0.05	-2.61	0.011
Failure to use turn signals (times)	3.33 ± 2.24	1.92 ± 1.58	1.4	1.0–1.8	7.12	<0.001
Incorrect use of turn signals (times)	0.61 ± 0.73	0.61 ± 0.61	0.00	-0.25 to 0.25	0.00	>0.9
Inadequate rear observation (times)	0.11 ± 0.31	0.05 ± 0.21	0.06	-0.04 to 0.16	1.27	0.2
Failure to observe the road ahead (times)	0.94 ± 0.85	0.61 ± 0.77	0.33	0.10–0.56	2.83	0.006
Disregarding traffic signals and road signs (times)	1.02 ± 1.00	0.73 ± 0.91	0.28	0.02–0.54	2.18	0.033
Improper halt (times)	1.73 ± 1.06	1.52 ± 1.11	0.22	-0.12 to 0.55	1.30	0.2
Improper lane positioning (times)	0.22 ± 0.52	0.11 ± 0.31	0.11	-0.03 to 0.25	1.54	0.13
Unsafe following and lateral distances (times)	0.0781 ± 0.2705	0.0156 ± 0.1250	0.06	0.00–0.12	2.05	0.045
Improper lane positioning during merging and cornering (times)	0.17 ± 0.38	0.06 ± 0.30	0.11	-0.01 to 0.23	1.84	0.070
Percentage of speeding in course (%)	4.1 ± 5.9	1.0 ± 2.2	3.1	1.5–4.6	4.00	<0.001
Average excess speed (km/h)	6.5 ± 5.9	2.6 ± 3.4	3.9	2.2–5.6	4.64	<0.001
Average turning speed at intersections (km/h)	14.8 ± 4.9	13.2 ± 3.5	1.6	0.19–2.9	2.28	0.026
Route deviation (times)	1.11 ± 1.22	0.41 ± 0.61	0.70	0.41–1.0	4.78	<0.001
Missed instructional signs (times)	0.42 ± 0.50	0.23 ± 0.43	0.19	0.05–0.32	2.83	0.006
Near collision within 1 m (times)	1.14 ± 0.96	0.80 ± 0.93	0.34	0.05–0.64	2.32	0.024
Accidents (times)	3.17 ± 2.73	1.17 ± 1.30	2.0	1.4–2.6	6.71	<0.001

Performance in the city-driving course also showed significant improvements following training. Patients exhibited better compliance with stop signs (p < 0.001) and demonstrated reductions in sudden braking (p = 0.006), failure to observe the road ahead (p = 0.006), disregarding traffic signals and road signs (p = 0.033), and unsafe following and lateral distance (p = 0.045). In addition, improvements were observed in successful parking (p = 0.011), failure to use turn signals (p < 0.001), and correct course navigation (p < 0.001). There were significant reductions in speeding violations (p < 0.001), average excess speed (p < 0.001), and average turning speed at intersections (p = 0.026), route deviating course (p < 0.001), missed instructional signs (p = 0.006), near collisions within 1 m (p = 0.024), and accidents (p < 0.001).

Multiple factor analysis of pre- and post-performance on the driving simulator

The results of the MFA indicated that the first three dimensions were retained (Appendix F). These three dimensions accounted for 33% of the overall variance, with individual contributions of 16.9%, 8.7%, and 7.4%, respectively (Appendix G).

Dimension 1 exhibited strong correlations with variables related to driving performance errors on the city-driving course. Notably, post-accident (0.771), pre-disregarding traffic signals and road signs (0.724), and pre-ignoring a stop sign (0.693) showed the highest correlations (Appendix H). Given these associations, dimension 1 was identified as representing errors during city driving.

Dimension 2 was predominantly associated with reaction task performance. Strong correlations were observed with post-reaction time (0.731), pre-reaction time (0.730), and pre-yellow lamp reaction time (0.719) (Appendix I). These findings indicate that dimension 2 captures variations in reaction time-related abilities.

Dimension 3 primarily reflected training-induced changes in driving performance. Positive correlations were found with post-training variables, including post-speeding on the course (0.707), post-unsafe following and lateral distance (0.706), and post-near collision within 1 m (0.507). In contrast, negative correlations were observed with pre-training variables such as pre-accidents (-0.489), pre-improper lane position (-0.469), and pre-improper halt (-0.355). These results suggest that dimension 3 is associated with performance improvements following training (Appendix J).

Results of Hierarchical Clustering on Principal Components

**Figure 3:**
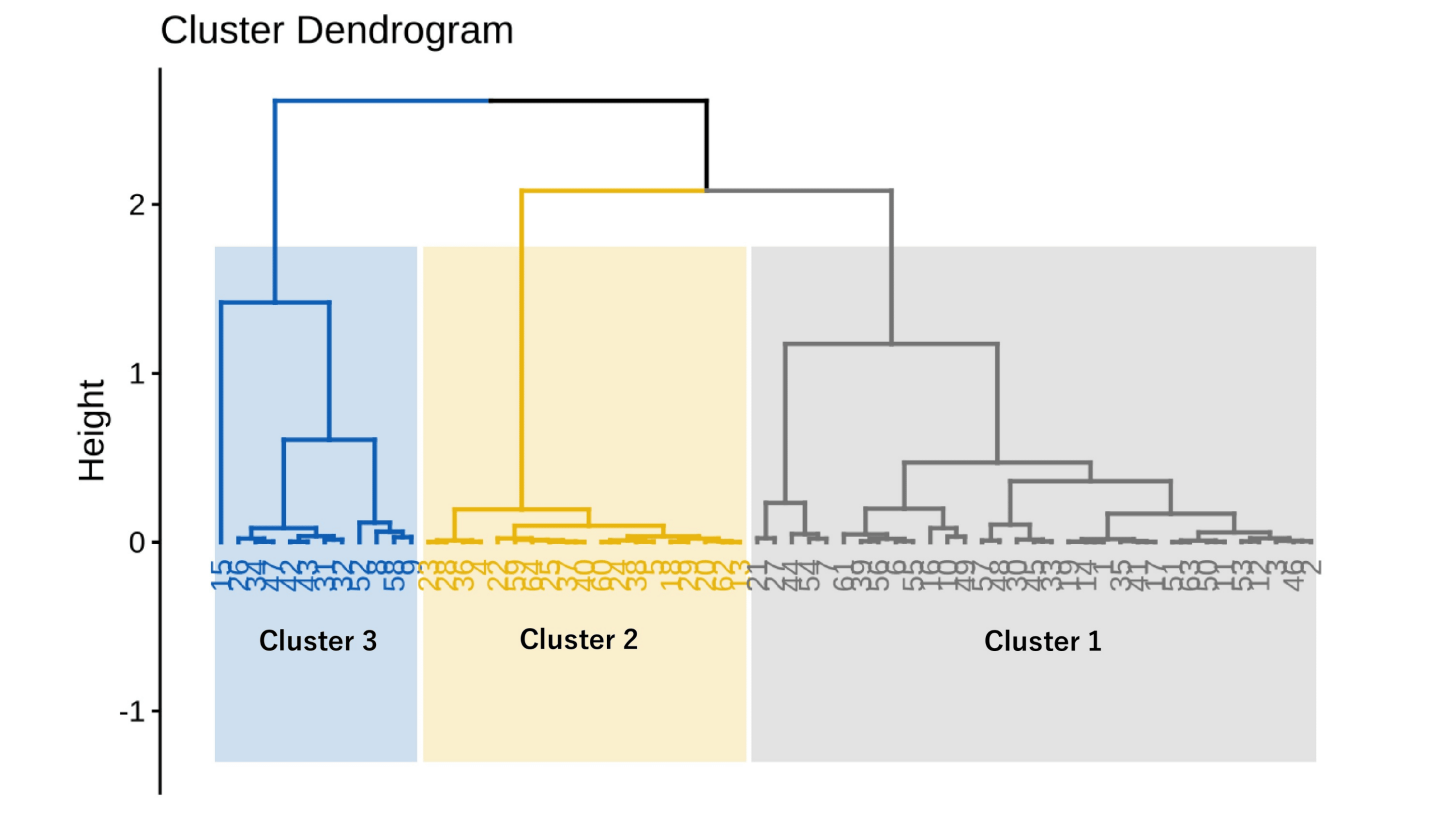
Cluster dendrogram from Hierarchical Clustering on Principal Components based on the first three dimensions of the multiple factor analysis.

**Figure 4:**
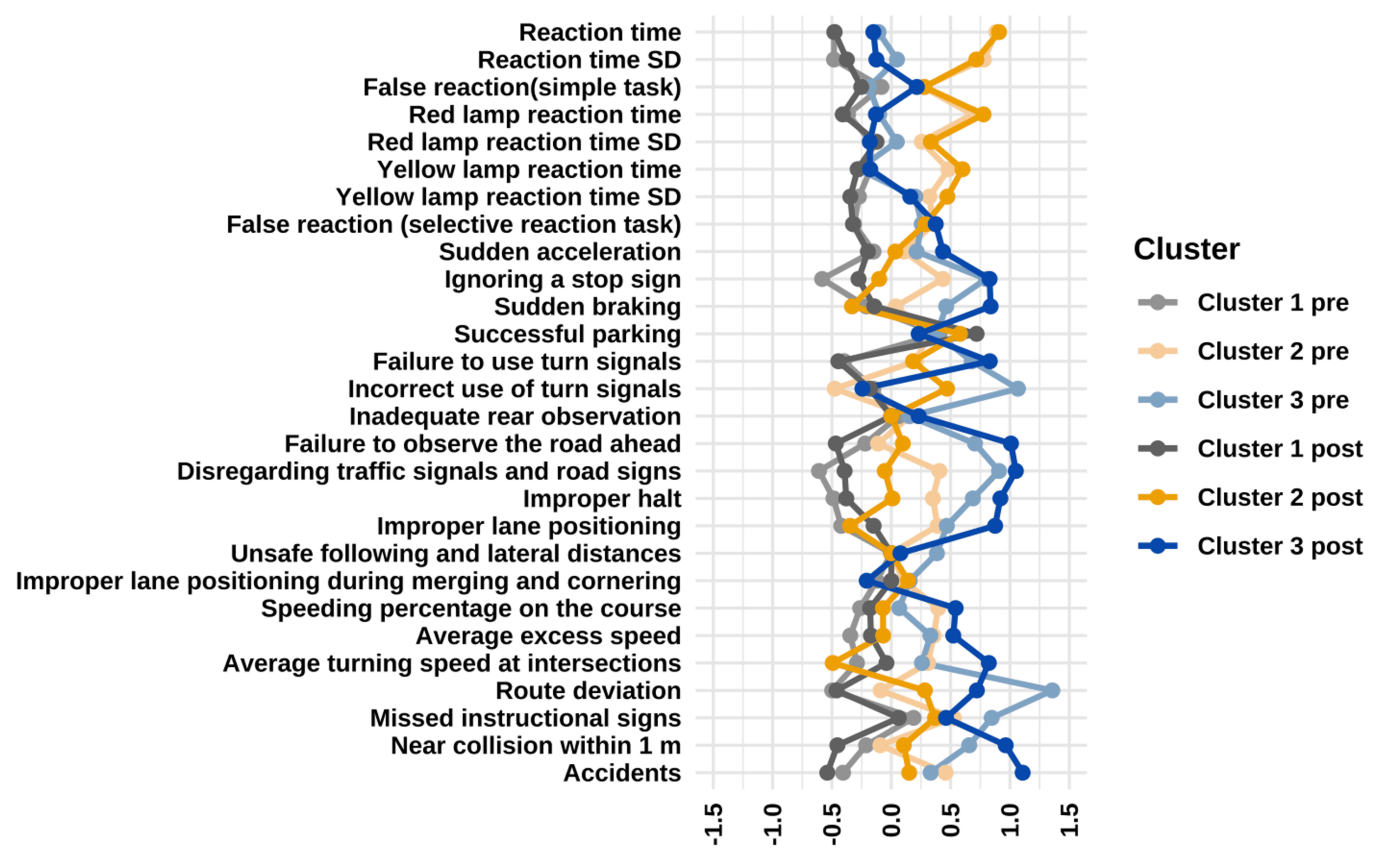
Parallel coordinate plot of driving simulator performance before and after training across the three clusters. This parallel coordinate plot visualizes pre- and post-training performance on all driving simulator metrics across the three identified clusters. Values represent standardized scores (higher values indicate poorer performance for most metrics except "successful parking"). Cluster 1 (n=32) maintained consistently better performance both before and after training. Cluster 2 (n=19) showed longer reaction times but demonstrated improvement in several city-driving metrics. Cluster 3 (n=13) exhibited the most pronounced difficulties, particularly in city-driving tasks, with limited improvement following training. This visualization highlights the distinctive patterns that characterize each cluster's response to driving simulator training.

The application of hierarchical clustering to the principal components of the first three dimensions resulted in the identification of three clusters (Figure 3). To visualize the distinctive performance patterns of each cluster, Figure 4 presents a parallel coordinate plot comparing pre- and post-training performance across all driving simulator metrics. This visualization reveals clear differences in baseline performance and improvement trajectories among the three clusters. Cluster 1 (gray/yellow lines) demonstrates consistently superior performance across most metrics both pre- and post-training. Cluster 2 (light blue/orange lines) shows challenges particularly in reaction time measures but exhibits improvements in several city-driving metrics following training. Cluster 3 (blue/dark blue lines) displays the most pronounced difficulties, especially in city-driving tasks, with limited improvement after training.

Cluster 1 (n = 32) exhibited considerable negative v-test values across several pre- and post-training variables, including pre-disregarding traffic signals and road signs (v-test = -4.88), pre-ignoring a stop sign (v-test = -4.66), post-accidents (v-test = -4.31), pre-route deviation (v-test = -3.99), pre-improper halt (v-test = -3.90), pre-reaction time SD (v-test = -3.86), pre-reaction time (v-test = -3.85), and post-reaction time (v-test = -3.82). These results indicate that patients in this cluster performed considerably better on these items than the overall average. In addition, post-successful parking (v-test = 2.26) had a positive v-test value, as this variable was scored based on successful parking (with a score of one), suggesting that patients in Cluster 1 had a higher successful parking rate than those in the other clusters. Based on these findings, Cluster 1 was defined as the group that consistently demonstrated superior performance in both reaction tasks and city driving courses at both the pre- and post-assessment stages (Appendix K).

Cluster 2 (n = 19) exhibited substantially longer and more variable reaction times, as indicated by high v-test values for variables such as post-reaction time (v-test = 4.72), pre-reaction time (v-test = 4.60), pre-reaction time SD (v-test = 4.06), post-red-lamp reaction time (v-test = 4.04), post-reaction time SD (v-test = 3.73), and pre-red-lamp reaction time (v-test = 3.55). These results indicate that patients in this cluster consistently had longer reaction times and greater variability in their responses than the overall average, both before and after training. In addition, several variables related to city-driving performance, such as post-incorrect use of turn signals (v-test = 2.44), pre-accidents (v-test = 2.38), pre-ignoring a stop sign (v-test = 2.26), pre-disregarding traffic signals and road signs (v-test = 2.11), pre-speeding percentage on the course (v-test = 2.06), and pre-improper lane positioning (v-test = 2.03), also had positive v-test values, indicating poorer performance in these areas. However, some variables, such as pre-incorrect use of turn signals (v-test = -2.48) and post-average turning speed at intersections (v-test = -2.56), exhibited negative v-test values, suggesting better performance than the overall average for these items. Furthermore, several city driving errors that had significantly positive v-test values in the pre-training phase, such as accidents, ignoring a stop sign, and disregarding traffic signals and road signs, were not statistically significant in the post-training phase. Based on these findings, Cluster 2 was defined as a group of patients who exhibited longer and more variable reaction times than the average but demonstrated potential for improvement in certain aspects of city driving performance after training (Appendix L).

Cluster 3 (n=13) demonstrated poor city-driving performance with high v-test values for pre-route deviation (v-test = 5.48), pre-unsafe following and lateral distance (v-test = 4.58), and post-accidents (v-test = 4.47), indicating persistent issues despite training. These results suggest that patients in this cluster exhibited a higher frequency of navigation errors, poor management of following and lateral distances, and a higher incidence of accidents than those in the other clusters. In addition, variables related to both pre- and post-training failures, such as ignoring a stop sign and disregarding traffic and road signs, also had positive v-test values, suggesting a persistent tendency to overlook traffic signals throughout the training period. Furthermore, variables associated with speed control, such as post-average turning speed at intersections (v-test = 3.32), post-speeding percentage on the course (v-test = 2.19), and post-average excess speed (v-test = 2.11), had highly positive v-test values, indicating that speeding and inadequate speed adjustments at intersections did not improve even after training. In addition, post-successful parking had a negative v-test value (v-test = -2.82), implying that the successful parking rate was lower in this cluster than in the other clusters. However, no significant v-test values were found for reaction time-related variables, indicating no distinct differences in reaction task performance compared to other clusters. Overall, more variables showed significant v-test values in the post-training assessment than in the pre-training assessment, demonstrating limited improvement after training. Cluster 3 was characterized by a high frequency of city-driving errors, particularly excessive speeding, inadequate distance management, and navigation errors. This cluster was defined as the group that showed minimal improvement after training, with many errors remaining unchanged despite intervention (Appendix M).

Demographic characteristics of clusters

The comparisons of background factors among the clusters are presented in Table 4. In the TMT-A, Cluster 1 had significantly shorter test times than Cluster 2 (p = 0.041). Similarly, in the TMT-B, Cluster 1 demonstrated significantly shorter test times compared to Cluster 2 (p = 0.003) and 3 (p = 0.031). In the KBDT, Cluster 1 had a higher IQ than Cluster 2 (p = 0.013). The results of the ROCFT showed that Cluster 1 achieved higher scores than Cluster 2 (p = 0.008) and 3 (p < 0.001). In the RBMT, Cluster 1 scored higher than Cluster 2 (p = 0.036). On the BADS, Cluster 1 had higher scores than Cluster 2 (p = 0.002) and Cluster 3 (p = 0.01). In addition, in the J-SDSA, the pass rate was significantly higher in Cluster 1 compared to Cluster 2 (p = 0.006) and Cluster 3 (p = 0.034). No statistically significant differences were observed in the other background factors.

**Table 4:** Demographic characteristics of the identified clusters. ^a^Data are presented as mean ± SD, n (%), or median (IQR).  ^b^Statistical tests: Kruskal-Wallis (continuous/ordinal) and Fisher's exact (categorical). ^c^Multiple comparisons: Mann-Whitney U tests (continuous and ordinal variables) and Fisher's exact tests (categorical variables), all with Bonferroni correction. ^d^Cerebral hemorrhage vs cerebral infarction. ^e^Cerebral infarction vs traumatic brain injury. ^f^Traumatic brain injury vs cerebral hemorrhage. FIM, Functional Independence Measure; BRS, Brunnstrom Recovery Stage; MMSE-J, Mini-Mental State Examination Japanese Version; TMT-J Part A and Part B, Trail Making Test Part A and Part B Japanese Version; BITC, Behavioral Inattention Test Conventional Subtests; KBDT, Kohs Block Design Test; ROCFT, Rey–Osterrieth Complex Figure Test; RBMT, Rivermead Behavioral Memory Test; BADS, Behavioral Assessment of the Dysexecutive Syndrome; J-SDSA, Stroke Drivers’ Screening Assessment Japanese Version; SD, standard deviation; IQR, interquartile range

Variable	Patients, n = 64	Cluster 1, n = 32^a^	Cluster 2, n = 19^a^	Cluster 3, n = 13^a^	Statistic^b^	p-Value^b^	Multiple comparisons^c^		
							1 vs 2	1 vs 3	2 vs 3
Age (years)	64 ± 13	62 ± 12	67 ± 13	65 ± 14	3.115	0.2	0.253	1.000	1.000
Sex						0.4	1.000	1.000	1.000
Female	12 (19%)	4 (13%)	5 (26%)	3 (23%)					
Male	52 (81%)	28 (88%)	14 (74%)	10 (77%)					
Diagnosis						0.072			
Cerebral hemorrhage	22 (34%)	13 (41%)	8 (42%)	1 (8%)			1.000^d^	0.210^d^	0.250^d^
Cerebral infarction	38 (59%)	17 (53%)	9 (47%)	12 (92%)			1.000^e^	0.096^e^	0.073^e^
Traumatic brain injury	4 (7%)	2 (6%)	2 (11%)	0 (0%)			1.000^f^	1.000^f^	1.000^f^
Lesion side					2.894	0.2	0.620	1.000	0.620
Right	30 (47%)	13 (41%)	12 (63%)	5 (38%)					
Left	34 (53%)	19 (59%)	7 (37%)	8 (62%)					
Onset-to-assessment time (days)	133 (32-267)	120 (30-197)	168 (33-367)	40 (22-259)	1.199	0.5	0.891	1.000	1.000
Interval between pre- and post-assessments (days)	9 (5-16)	10 ± 8	14 ± 11	14 ± 13	1.323	0.5	0.876	1.000	1.000
FIM									
Motor function score	88 ± 3	89 ± 3	88 ± 3	87 ± 4	2.481	0.3	0.627	0.600	1.000
Cognitive function score	34 ± 2	34 ± 2	34 ± 2	33 ± 2	1.926	0.4	1.000	0.636	0.705
Total score	122 ± 4	123 ± 4	122 ± 3	120 ± 4	4.882	0.087	0.756	0.109	0.735
BRS									
Upper extremity	6 (5-6)	6 (5-6)	6 (6-6)	6 (6-6)	2.063	0.8	0.528	0.978	1.000
Hand/fingers	6 (6-6)	6 (6-6)	6 (6-6)	6 (6-6)	1.595	0.8	0.636	1.000	1.000
Lower extremity	6 (6-6)	6 (6-6)	6 (6-6)	6 (6-6)	0.824	0.6	0.399	0.547	0.940
MMSE-J total score	28 ± 2	29 ± 1	28 ± 2	28 ± 3	1.818	0.4	0.534	1.000	1.000
TMT-J Part A (s)	77 ± 36	66 ± 32	90 ± 34	85 ± 42	6.490	0.039	0.041	0.3	0.659
TMT-J Part B (s)	129 ± 65	99 ± 44	158 ± 69	157 ± 75	12.895	0.002	0.003	0.031	0.893
BIT total score	142 ± 4	142 ± 5	141 ± 5	142 ± 3	1.240	0.5	0.420	0.338	0.786
KBDT IQ score	89 ± 19	96 ± 19	79 ± 11	84 ± 19	8.682	0.013	0.013	0.202	0.514
ROCFT copy score	33.55 ± 3.42	35.13 ± 1.86	33.11 ± 4.43	31.85 ± 3.69	16.162	<0.001	0.008	< 0.001	0.144
RBMT total profile score	19 ± 4	20 ± 3	18 ± 3	17 ± 5	8.341	0.015	0.074	0.036	0.418
BADS total score	16 ± 4	18 ± 3	15 ± 4	15 ± 3	12.718	0.002	0.009	0.01	0.938
J-SDSA prediction of fitness to drive						0.001	0.006	0.034	1.000
Failed	19 (30%)	3 (10%)	10 (53%)	6 (46%)					
Passed	45 (70%)	29 (90%)	9 (47%)	7 (54%)					

## Discussion

This study aimed to classify patients with ABI based on their driving simulator performance using clustering analysis and to explore the relationship between identified performance patterns and associated cognitive and demographic factors. Three distinct clusters were identified, each characterized by different baseline performance patterns and responses to training: Cluster 1 demonstrated consistently superior performance across cognitive and driving assessments, Cluster 2 showed specific deficits in reaction time but capacity for improvement in practical driving skills, and Cluster 3 exhibited persistent difficulties with limited training benefits. These distinct patterns highlight the heterogeneity in driving rehabilitation potential among patients with ABI and underscore the importance of individualized assessment approaches. To our knowledge, this is the first study to systematically investigate the relationship between driving simulation performance and background factors in patients with ABI.

Patients in Cluster 1 exhibited superior cognitive profiles, including strong attentional function, cognitive flexibility, visuospatial abilities, visual construction skills, memory, and executive function [27, 29-33], which likely contributed to their consistently high driving performance. These findings align with previous studies that highlight the importance of neuropsychological assessments in predicting driving ability [37]. This group was considered capable of efficiently resuming driving using standard training protocols.

Patients in Cluster 2 demonstrated instability in their reaction task performance, characterized by longer reaction times and greater variability. However, they showed improvements in the city-driving course after training, including a reduction in signal errors and better adherence to traffic rules. These findings are consistent with the results of previous studies on driving simulator training conducted with patients with traumatic brain injury or mild cognitive impairment [10,38]. Specifically, prior research has reported improvements in driving errors such as speeding, improper lane positioning, disregarding traffic signs, and hazardous driving behaviors. Similarly, in this study, improvements in city-driving performance were observed among patients with ABI, reinforcing previous findings. The improvements observed in Cluster 2 may have been influenced by memory abilities, as suggested by the RBMT scores. Although no significant difference in RBMT scores was found between Clusters 1 and 2, a significant difference was observed between Clusters 1 and 3. This suggests that memory may have played a role in the effectiveness of the training. Previous research has shown that cognitive functions such as attention, information processing speed, working memory, and long-term memory are crucial for learning effects during training [39,40]. Furthermore, studies have reported that older adults with memory impairments often struggle to benefit from training programs based on learning strategies [41]. In this study, patients in Cluster 2 appeared to have learned from their previous mistakes in the city-driving course, particularly those related to route navigation, traffic signs, and hazardous events, and retained this knowledge, which likely contributed to the observed training effects. In addition, the feedback provided by them may have helped patients adopt appropriate driving behaviors, enhance their self-awareness, and apply these improvements in practice. Moreover, even if operational abilities such as reaction time and its variability decline - consistent with Michon’s model of driving behavior [42] - patients without memory impairments may still recall past mistakes and experiences and adopt strategic compensatory actions, known as strategic-level behaviors, to improve their driving performance. For example, consciously adopting driving strategies to compensate for delayed reactions and practicing learned techniques may enhance training outcomes.

Driving errors common to patients in Clusters 2 and 3 included accidents, ignoring a stop sign, disregarding traffic and road signs, excessive speeding, improper lane positioning, and the incorrect use of turn signals. These errors have also been identified in previous studies [43-47] as being associated with cognitive profiles, which demonstrated considerable differences in this study.

Patients in Cluster 3 demonstrated persistent driving errors that showed minimal improvement despite training. Their performance was characterized by significant difficulties in multiple domains, particularly in city-driving tasks that demand integrated cognitive functions. The limited improvement observed in this group aligns with their poorer cognitive profiles, especially in memory, visuospatial ability, and executive function domains - cognitive areas that are critical for developing compensatory strategies and learning from training. These specific cognitive deficits have been associated with driving errors in several previous studies [44,46,48-52]. Therefore, individualized programs targeting these cognitive functions, such as visual attention training and rehabilitation focused on enhancing problem-solving skills [53], are likely to be beneficial.

Clinical implications

The identification of distinct response patterns to driving simulator training has several important clinical implications. For rehabilitation practitioners, our findings suggest that comprehensive neuropsychological assessment before simulator training may help predict not only baseline performance but also the potential for improvement. Specifically, Patients with profiles similar to Cluster 1 may benefit from abbreviated simulator training protocols, potentially allowing earlier progression to on-road assessment. For patients resembling Cluster 2 (with relatively preserved memory but slower processing speed), training approaches that emphasize compensatory strategies and provide extended practice opportunities may be most beneficial. These patients may particularly benefit from targeted interventions to improve reaction times and hazard perception. Patients with cognitive profiles similar to Cluster 3 may require more intensive cognitive rehabilitation targeting specific domains (memory, executive function, and visuospatial abilities) before or concurrent with driving simulator training. Alternatively, these patients might benefit from exploring transportation alternatives if safe driving remains challenging despite intervention.

These tailored approaches could optimize rehabilitation resources while maximizing each patient's potential for successful driving resumption.

This study had some limitations worth noting. First, it was conducted at a single facility, which may have introduced selection bias. In addition, the sample was notably unbalanced, with 81% of participants being male and only 7% having traumatic brain injuries. These selective patient characteristics may limit the generalizability of our findings. Second, the cross-sectional design restricted the ability to establish a direct causal relationship between cognitive profiles and driving simulator performance. For instance, although significant differences in cognitive profiles were observed, it remains unclear how these differences directly impact driving performance. Several factors beyond cognitive and motor abilities may have influenced our findings. Psychological factors such as motivation, anxiety, and confidence likely affected both training engagement and performance outcomes. Participants in Cluster 2, who showed improvement despite initial reaction time deficits, might have demonstrated higher motivation or less simulator-related anxiety than those in Cluster 3. Additionally, fatigue could have differentially affected performance across clusters, particularly in tasks requiring sustained attention. Pre-injury driving experience and habits, though not systematically assessed in this study, may also have influenced baseline performance and adaptation to the simulator environment. Further research, including intervention-based and longitudinal studies, is necessary to clarify this causal relationship. Third, the study's sample size was limited. Although 64 patients were included, the number of participants in each cluster was too small for robust statistical analysis. In particular, Cluster 3 had only 13 participants, which may have affected the statistical interpretation and generalizability of the findings. Future studies with larger sample sizes are needed to enhance the reliability and applicability of the results. Finally, although nine training sessions were conducted to improve driving performance, the duration, frequency, and instructional methods may have been insufficient for some patients. This could have contributed to the limited improvements observed in the driving simulator performance. Future studies should consider extending the training period based on individual patient needs and implementing adaptive training programs tailored to specific cognitive profiles to maximize effectiveness.

## Conclusions

This study assessed driving simulator performance before and after training and classified the driving performance of patients with ABI through clustering analysis using MFA and HCPC. The findings emphasize the importance of tailoring training programs to individual patient characteristics by identifying driving performance patterns based on cognitive profiles. Specifically, evaluating cognitive profiles to identify weaknesses in driving performance and designing targeted training programs could enhance the effectiveness of rehabilitation. These results provide valuable data for developing intervention strategies to support the resumption of driving in patients with ABI. Future research should focus on validating these clusters in larger samples and developing specific cognitive training protocols targeting the deficits identified in each cluster. Additionally, investigating the efficacy of personalized rehabilitation pathways based on these cluster profiles could advance clinical practice in driving rehabilitation after ABI.

## Figures and Tables

**Figure 5:**
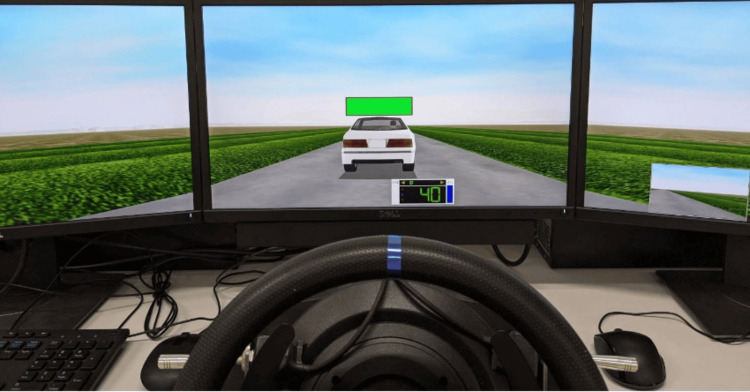
Simple reaction task The patients were required to release the accelerator pedal when the green lamp of the vehicle in front illuminated.

**Figure 6:**
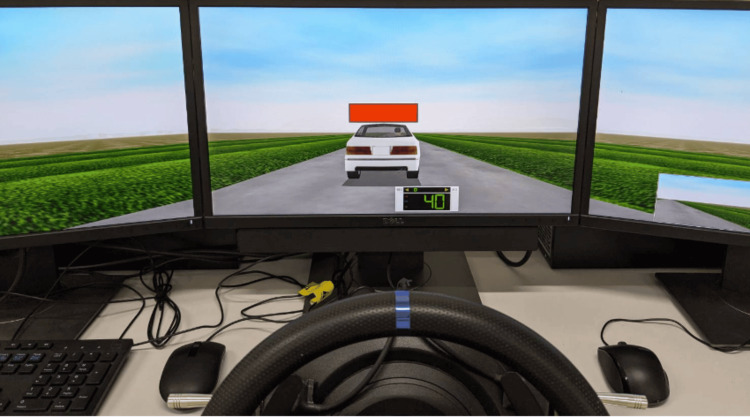
Selective reaction task - red lamp Immediately step on the brake pedal from a depressed accelerator pedal position.

**Figure 7:**
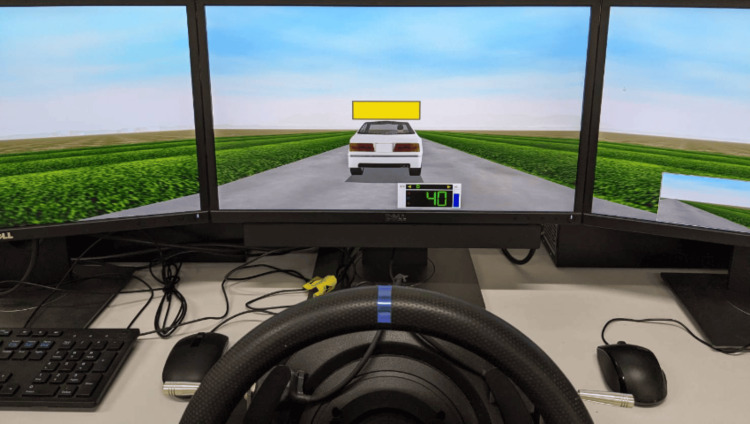
Selective reaction task - yellow lamp Release the accelerator pedal from a depressed position.

**Figure 8:**
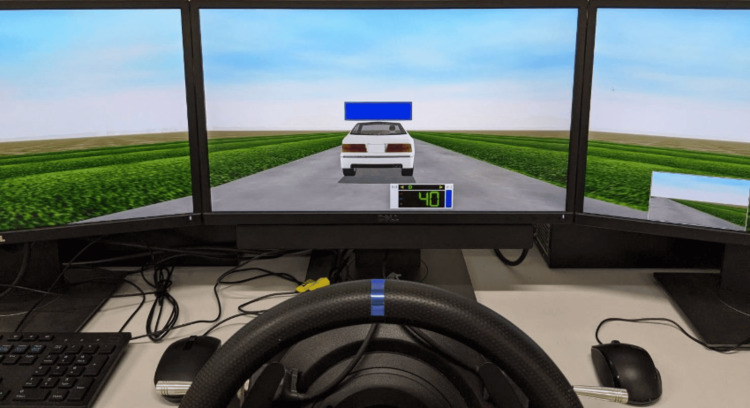
Selective reaction task - blue lamp Keep pressing the accelerator pedal.

**Figure 9:**
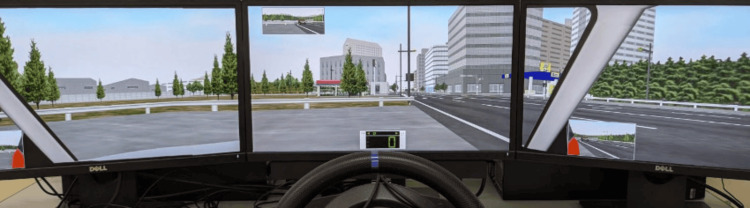
City-driving course The patients drive through a virtual, simulated city environment.

**Figure 10:**
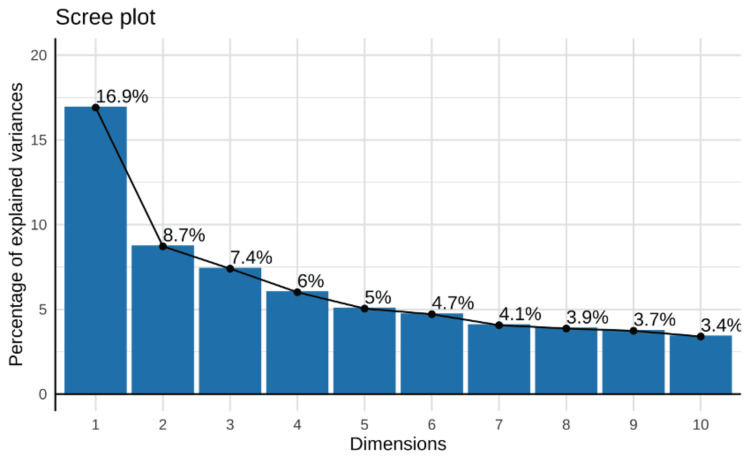
Scree plot showing the percentage of explained variance by dimension in the multiple factor analysis. The eigenvalues for the first 10 dimensions exceeded 1, indicating that each of these dimensions explained a significant proportion of the variance in the pre- and post-training driving simulator variables. An elbow point was observed between the first and second dimensions of the scree plot, leading to the retention of the first three dimensions. The retained dimensions were defined as follows: Dimension 1 as city-driving performance errors, Dimension 2 as reaction task performance, and Dimension 3 as training-induced changes. These definitions were based on the significant contributions of key variables within each dimension.

## Appendices

Appendix A 

Appendix B

Appendix C

Appendix D 

Appendix E

Appendix F 

Appendix G

**Table 5:** Eigenvalue, percentage of variance, and cumulative percentage of variance by dimension in the multiple factor analysis.

Component	Eigenvalue	Percentage of variance	Cumulative percentage of variance
comp 1	5.483	16.906	16.906
comp 2	2.828	8.719	25.625
comp 3	2.400	7.401	33.026
comp 4	1.950	6.013	39.039
comp 5	1.637	5.047	44.086
comp 6	1.528	4.713	48.798
comp 7	1.319	4.067	52.866
comp 8	1.256	3.872	56.737
comp 9	1.211	3.735	60.472
comp 10	1.103	3.400	63.872
comp 11	0.963	2.970	66.842
comp 12	0.884	2.725	69.567
comp 13	0.859	2.648	72.214
comp 14	0.801	2.470	74.684
comp 15	0.683	2.105	76.789
comp 16	0.655	2.019	78.807
comp 17	0.610	1.882	80.689
comp 18	0.567	1.747	82.437
comp 19	0.553	1.705	84.142
comp 20	0.532	1.641	85.783
comp 21	0.487	1.501	87.284
comp 22	0.413	1.274	88.558
comp 23	0.394	1.214	89.772
comp 24	0.371	1.143	90.915
comp 25	0.329	1.014	91.929
comp 26	0.296	0.913	92.842
comp 27	0.257	0.792	93.634
comp 28	0.233	0.718	94.353
comp 29	0.220	0.677	95.030
comp 30	0.197	0.606	95.636
comp 31	0.172	0.532	96.168
comp 32	0.151	0.464	96.632
comp 33	0.142	0.437	97.069
comp 34	0.131	0.405	97.473
comp 35	0.102	0.315	97.788
comp 36	0.093	0.287	98.075
comp 37	0.089	0.275	98.350
comp 38	0.080	0.247	98.597
comp 39	0.073	0.225	98.822
comp 40	0.069	0.212	99.034
comp 41	0.056	0.172	99.206
comp 42	0.043	0.133	99.338
comp 43	0.039	0.122	99.460
comp 44	0.034	0.105	99.565
comp 45	0.030	0.093	99.658
comp 46	0.028	0.085	99.743
comp 47	0.022	0.069	99.812
comp 48	0.021	0.064	99.875
comp 49	0.014	0.042	99.918
comp 50	0.007	0.022	99.940
comp 51	0.006	0.017	99.958
comp 52	0.005	0.015	99.972
comp 53	0.003	0.010	99.982
comp 54	0.003	0.009	99.991
comp 55	0.002	0.007	99.998
comp 56	0.001	0.002	100.000

Appendix H

**Table 6:** Correlation coefficients and p-values for variables in Dimension 1 of the multiple factor analysis. The correlation coefficients indicate the strength and direction of the relationship between each variable and Dimension 1, with only those with p-values < 0.05 included. Positive values indicate a positive association, and negative values indicate a negative association.

Variable	Correlation	p-Value
Post-accidents	0.771	<0.001
Pre-disregarding traffic signals and road signs	0.724	<0.001
Pre-ignoring a stop sign	0.693	<0.001
Post-failure to observe the road ahead	0.657	<0.001
Pre-improper stopping position	0.645	<0.001
Post-disregarding traffic signals and road signs	0.621	<0.001
Post-near collision within 1 m	0.604	<0.001
Post-failure to use turn signals	0.603	<0.001
Post-route deviation	0.596	<0.001
Pre-route deviation	0.563	<0.001
Pre-improper lane positioning	0.563	<0.001
Pre-accidents	0.550	<0.001
Post-ignoring a stop sign	0.511	<0.001
Pre-unsafe following and lateral distance	0.500	<0.001
Pre-failure to use turn signals	0.500	<0.001
Pre-reaction time SD	0.479	<0.001
Post-false reaction (selective reaction task)	0.473	<0.001
Post-improper halt	0.465	<0.001
Pre-missed instructional signs	0.460	<0.001
Post-missed instructional signs	0.457	<0.001
Post-improper lane positioning	0.416	<0.001
Pre-near collision within 1 m	0.392	0.001
Post-reaction time	0.384	0.002
Pre-reaction time	0.376	0.002
Pre-failure to observe the road ahead	0.374	0.002
Post-red lamp reaction time	0.368	0.003
Pre-false reaction (selective reaction task)	0.365	0.003
Pre-average turning speed at intersections	0.345	0.005
Post-reaction time SD	0.332	0.007
Pre-average excess speed	0.327	0.008
Pre-incorrect use of turn signals	0.324	0.009
Post-yellow lamp reaction time SD	0.316	0.011
Pre-sudden braking	0.314	0.011
Pre-red lamp reaction time	0.313	0.012
Post-average turning speed at intersections	0.284	0.023
Post-speeding percentage on the course	0.271	0.031
Post-sudden acceleration	0.247	0.049
Post-successful parking	-0.479	<0.001

Appendix I 

**Table 7:** Correlation coefficients and p-values for variables in Dimension 2 of the multiple factor analysis. The correlation coefficients indicate the strength and direction of the relationship between each variable and Dimension 2, with only those with p-values < 0.05 included. Positive values indicate a positive association, and negative values indicate a negative association.

Variable	Correlation	p-Value
Post-reaction time	0.731	<0.001
Pre-reaction time	0.730	<0.001
Post-yellow lamp reaction time	0.719	<0.001
Pre-red lamp reaction time	0.636	<0.001
Pre-yellow lamp reaction time	0.618	<0.001
Post-yellow lamp reaction time SD	0.612	<0.001
Pre-reaction time SD	0.606	<0.001
Post-red lamp reaction time	0.529	<0.001
Pre-yellow lamp reaction time SD	0.465	<0.001
Post-reaction time SD	0.461	<0.001
Pre-red lamp reaction time SD	0.369	0.003
Post-red lamp reaction time SD	0.268	0.032
Post-unsafe following and lateral distances	0.246	0.050
Post-ignoring a stop sign	-0.252	0.045
Pre-missed instructional signs	-0.256	0.041
Post-sudden braking	-0.277	0.027
Pre-incorrect use of turn signals	-0.324	0.009
Post-inadequate rear observation	-0.342	0.006
Pre-route deviation	-0.425	<0.001
Post-improper lane positioning	-0.478	<0.001

Appendix J 

**Table 8:** Correlation coefficients and p-values for variables in Dimension 3 of the multiple factor analysis. The correlation coefficients indicate the strength and direction of the relationship between each variable and Dimension 3, with only those with p-values < 0.05 included. Positive values indicate a positive association, and negative values indicate a negative association.

Variable	Correlation	p-Value
Post-speeding percentage on the course	0.707	<0.001
Post-unsafe following and lateral distances	0.706	<0.001
Post-near collision within 1 m	0.507	<0.001
Post-average excess speed	0.502	<0.001
Post-failure to observe the road ahead	0.400	0.001
Post-disregarding traffic signals and road signs	0.359	0.004
Post-missed instructional signs	0.352	0.004
Post-yellow lamp reaction time SD	0.340	0.006
Pre-unsafe following and lateral distances	0.315	0.011
Pre-successful parking	0.269	0.031
Pre-near collision within 1 m	-0.247	0.049
Pre-inadequate rear observation	-0.279	0.026
Pre-failure to use turn signals	-0.279	0.026
Pre-ignoring a stop sign	-0.283	0.024
Pre-speeding percentage on the course	-0.311	0.012
Pre-average excess speed	-0.331	0.008
Pre-improper halt	-0.355	0.004
Post-red lamp reaction time SD	-0.434	<0.001
Pre-improper lane positioning	-0.469	<0.001
Pre-accidents	-0.489	<0.001

Appendix K 

**Table 9:** Hierarchical Clustering on Principal Components—description of quantitative variables by Cluster 1. The v-test compares the mean of each variable within the cluster with the overall mean. Negative values indicate that the cluster mean is lower than the overall mean, whereas positive values indicate that the cluster mean is higher. The v-test values follow Student’s t-distribution, and values > |1.96| correspond to p-values < 0.05, indicating statistical significance.

Variable	v-Test	Mean in category	Overall mean	SD in category	Overall SD	p-Value
Post-successful parking	2.260	0.719	0.578	0.450	0.494	0.024
Post-false reaction (simple reaction task)	-2.027	0.094	0.625	0.291	2.080	0.043
Pre-speeding percentage on the course	-2.097	2.572	4.126	4.470	5.879	0.036
Pre-yellow lamp reaction time SD	-2.180	0.159	0.180	0.055	0.077	0.029
Post-ignoring a stop sign	-2.209	0.688	1.016	0.845	1.179	0.027
Post-yellow lamp reaction time	-2.269	0.686	0.722	0.105	0.126	0.023
Pre-unsafe following and lateral distances	-2.311	0.000	0.078	0.000	0.268	0.021
Pre-average turning speed at intersections	-2.314	13.366	14.779	2.952	4.847	0.021
Pre-false reaction (selective reaction task)	-2.508	4.094	7.047	7.481	9.345	0.012
Post-false reaction (selective reaction task)	-2.585	2.125	3.172	3.018	3.214	0.010
Post-yellow lamp reaction time SD	-2.762	0.128	0.149	0.043	0.060	0.006
Pre-average excess speed	-2.766	4.502	6.529	5.063	5.815	0.006
Pre-red lamp reaction time	-2.916	1.005	1.094	0.223	0.240	0.004
Post-reaction time SD	-2.996	0.063	0.083	0.045	0.054	0.003
Post-improper halt	-3.033	1.094	1.516	0.947	1.104	0.002
Post-disregarding traffic signals and road signs	-3.150	0.375	0.734	0.650	0.906	0.002
Pre-failure to use turn signals	-3.181	2.438	3.328	1.676	2.222	0.001
Post-missed instructional signs	-3.220	0.062	0.234	0.242	0.424	0.001
Pre-accidents	-3.252	2.062	3.172	1.391	2.707	0.001
Post-red lamp reaction time	-3.270	0.888	0.983	0.212	0.231	0.001
Pre-improper lane positioning	-3.375	0.000	0.219	0.000	0.514	<0.001
Post-failure to use turn signals	-3.568	1.219	1.922	0.960	1.564	<0.001
Post-near collision within 1 m	-3.633	0.375	0.797	0.696	0.922	<0.001
Post-route deviation	-3.689	0.125	0.406	0.331	0.605	<0.001
Post-failure to observe the road ahead	-3.740	0.250	0.609	0.500	0.763	<0.001
Pre-missed instructional signs	-3.767	0.188	0.422	0.390	0.494	<0.001
Post-reaction time	-3.821	0.393	0.424	0.050	0.065	<0.001
Pre-reaction time	-3.852	0.410	0.459	0.050	0.100	<0.001
Pre-reaction time SD	-3.863	0.077	0.117	0.036	0.082	<0.001
Pre-improper halt	-3.900	1.219	1.734	0.856	1.049	<0.001
Pre-route deviation	-3.986	0.500	1.109	0.612	1.213	<0.001
Post-accidents	-4.314	0.469	1.172	0.706	1.294	<0.001
Pre-ignoring a stop sign	-4.663	0.844	1.656	0.795	1.383	<0.001
Pre-disregarding traffic signals and road signs	-4.876	0.406	1.016	0.551	0.992	<0.001

Appendix L 

**Table 10:** Hierarchical Clustering on Principal Components—description of quantitative variables by Cluster 2. The v-test compares the mean of each variable within the cluster with the overall mean. Negative values indicate that the cluster mean is lower than the overall mean, whereas positive values indicate that the cluster mean is higher. The v-test values follow Student’s t-distribution, and values > |1.96| correspond to p-values < 0.05, indicating statistical significance.

Variable	v-test	Mean in category	Overall mean	SD in category	Overall SD	p-value
Post-reaction time	4.719	0.484	0.424	0.061	0.065	<0.001
Pre-reaction time	4.600	0.548	0.459	0.106	0.100	<0.001
Pre-reaction time SD	4.056	0.181	0.117	0.096	0.082	<0.001
Post-red lamp reaction time	4.039	1.164	0.983	0.202	0.231	<0.001
Post-reaction time SD	3.726	0.122	0.083	0.059	0.054	<0.001
Pre-red lamp reaction time	3.554	1.259	1.094	0.200	0.240	<0.001
Post-yellow lamp reaction time	3.120	0.798	0.722	0.119	0.126	0.002
Pre-yellow lamp reaction time	2.507	0.886	0.797	0.159	0.182	0.012
Post-yellow lamp reaction time SD	2.455	0.178	0.149	0.062	0.060	0.014
Post-incorrect use of turn signals	2.443	0.895	0.609	0.640	0.603	0.015
Pre-accidents	2.380	4.421	3.172	3.574	2.707	0.017
Pre-ignoring a stop sign	2.263	2.263	1.656	1.250	1.383	0.024
Pre-disregarding traffic signals and road signs	2.108	1.421	1.016	0.815	0.992	0.035
Pre-speeding percentage on the course	2.057	6.470	4.126	8.256	5.879	0.040
Pre-improper lane positioning	2.028	0.421	0.219	0.674	0.514	0.043
Pre-incorrect use of turn signals	-2.478	0.263	0.609	0.440	0.721	0.013
Post-average turning speed at intersections	-2.565	11.499	13.218	2.510	3.457	0.010

Appendix M 

**Table 11:** Hierarchical Clustering on Principal Components—description of quantitative variables by Cluster 3. The v-test compares the mean of each variable within the cluster with the overall mean. Negative values indicate that the cluster mean is lower than the overall mean, whereas positive values indicate that the cluster mean is higher. The v-test values follow Student’s t-distribution, and values > |1.96| correspond to p-values < 0.05, indicating statistical significance.

Variable	v-Test	Mean in category	Overall mean	SD in category	Overall SD	p-Value
Pre-route deviation	5.482	2.769	1.109	1.187	1.213	<0.001
Pre-unsafe following and lateral distances	4.577	0.385	0.078	0.487	0.268	<0.001
Post-accidents	4.471	2.615	1.172	1.443	1.294	<0.001
Pre-incorrect use of turn signals	4.311	1.385	0.609	0.836	0.721	<0.001
Post-disregarding traffic signals and road signs	4.239	1.692	0.734	1.066	0.906	<0.001
Post-failure to observe the road ahead	4.073	1.385	0.609	0.836	0.763	<0.001
Post-near collision within 1 m	3.894	1.692	0.797	0.991	0.922	<0.001
Post-improper halt	3.713	2.538	1.516	1.151	1.104	<0.001
Pre-disregarding traffic signals and road signs	3.666	1.923	1.016	1.071	0.992	<0.001
Post-improper lane positioning	3.534	0.385	0.109	0.487	0.312	<0.001
Post-inadequate rear observation	3.486	0.231	0.047	0.421	0.211	<0.001
Pre-missed instructional signs	3.443	0.846	0.422	0.361	0.494	<0.001
Post-sudden braking	3.381	0.923	0.375	0.828	0.650	<0.001
Post-failure to use turn signals	3.353	3.231	1.922	1.804	1.564	<0.001
Post-ignoring a stop sign	3.345	2.000	1.016	1.569	1.179	<0.001
Post-average turning speed at intersections	3.320	16.082	13.218	3.840	3.457	<0.001
Pre-failure to stop at a stop sign	3.225	2.769	1.656	1.476	1.383	0.001
Post-route deviation	2.913	0.846	0.406	0.769	0.605	0.004
Pre-failure to observe the road ahead	2.848	1.538	0.938	1.151	0.845	0.004
Pre-improper halt	2.777	2.462	1.734	1.151	1.049	0.005
Pre-failure to use turn signals	2.737	4.846	3.328	2.381	2.222	0.006
Pre-near collision within 1 m	2.652	1.769	1.141	1.187	0.950	0.008
Post-speeding percentage on the course	2.190	2.233	1.049	3.551	2.167	0.029
Post-missed instructional signs	2.149	0.462	0.234	0.499	0.424	0.032
Post-average excess speed	2.110	4.442	2.645	3.172	3.413	0.035
Post-unsafe following and lateral distances	1.981	0.077	0.016	0.266	0.124	0.048
Post-successful parking	-2.819	0.231	0.578	0.421	0.494	0.005
